# The Immunologic Properties of Bone Morphogenic Protein Receptor IB Positive Subpopulation before and after Osteogenic Differentiation in Mouse Dermis

**DOI:** 10.1371/journal.pone.0161785

**Published:** 2016-08-23

**Authors:** Jin-Guang He, Ting-Liang Wang, Tao Wang, Hua Xu, Yi Zhang, Jia-Sheng Dong

**Affiliations:** Department of Plastic and Reconstructive Surgery, Shanghai ninth People’s Hospital, Shanghai JiaoTong University School of Medicine, Shanghai, China; Second University of Naples, ITALY

## Abstract

We have previously reported that human dermal bone morphogenic protein receptor (BMPR) IB positive subpopulation had a high osteogenic differentiation potential and may be a promising cell source for allogeneic bone tissue engineering. In this study, the immunologic properties of dermal BMPR-IB^+^ subpopulation before and after osteogenic differentiation were reported. The results confirmed that dermal BMPR-IB^+^ cells possessed a similar osteogenic differentiation potential with bone marrow mesenchymal stromal cells in a mouse model. Furthermore, the expression of immune rejection-related surface antigens such as major histocompatibility class II and co-stimulatory proteins (CD40, CD80, and CD86) were absent on dermal BMPRIB^+^ cells. Dermal BMPRIB^+^ cells elicited no proliferation of allogeneic splenocytes and suppressed the proliferation of stimulated immune cells. Interestingly, osteogenic differentiation *in vitro* had no adverse effect on the immunological features of these cells. Most importantly, inducible NO synthase (iNOS) was involved in immunoregulatory effects by undifferentiated BMPRIB^+^ fibroblasts, whereas indoleamine 2,3-dioxygenase (IDO) activity was related to mediating immunomodulatory function by osteogenic differentiated BMPRIB^+^ fibroblasts. In conclusion, dermal BMPRIB^+^ cells have a low immunogenicity and possess immunosuppressive capacity before and after osteogenic differentiation *in vitro*, which would facilitate the allotransplantation in the future. However, mechanisms mediating immunoregulatory property between undifferentiated and osteogenic differentiated BMPRIB^+^ fibroblasts may be different and need further investigation.

## Introduction

Cell-based therapy has been an attractive new option in skeletal regenerative medicine [1–3].Skin dermis is reported to contain progenitors with osteogenic, adipogenic, and chondrogenic differentiation potential *in vitro* [4, 5]. In our previous study, we identified a subpopulation of bone morphogenetic protein receptor IB (BMPR-IB) positive cells in human dermis. They possessed a high osteogenic differentiation potential and could be easily isolated in large numbers. Therefore, dermal BMPR-IB^+^ subpopulation maybe a promising mesenchymal stem cell (MSC)-like cell source for cell based bone defect repair [3].

In order to provide “off-the shelf” cell therapies and readily available tissue engineered products, it is also essential to utilize a pool of well characterized dermal BMPR-IB^+^ cells from allogeneic sources generating enough osteoblast-like cells for clinical transplantation[6]. Nevertheless, whether allogeneic dermal BMPR-IB^+^ cells would lead to rejection by host immune system have not been investigated up to date. Furthermore, the potential clinical applications generally require allogeneic dermal BMPR-IB^+^ cells differentiate into mature osteoblasts or osteocytes before transplantation. It is not clear yet that the immunological characteristics of dermal BMPR-IB^+^ cells would be stable when exposed to osteogenic differentiation.

It has been demonstrated that bone marrow mesenchymal stromal cells (BMSCs) do not elicit allogeneic lymphocyte proliferation and have the potential to inhibit allogeneic mixed lymphocyte reactions *in vitro*[6–9].Moreover, in an animal model, intravenous administration of allogeneic BMSCs to major histocompatibility class (MHC) mismatched recipient led to prolonged skin graft survival[10]. Immunosuppressive capacity is thus believed to be the primitive function of mesenchymal stem cells [11]. In addition, several studies demonstrated that BMSCs maintained their immunomodulatory effect after differentiating into various mesenchymal lineages [12,13].

However, the underlying mechanisms by which BMSCs mediate immune suppressive effect are not clear. Some reports have shown that the direct cell-cell contact played an important role in mediating the immunosuppressive effects of BMSCs [14]. Moreover, a number of soluble factors such as, prostaglandin E2, transforming growth factor b1 (TGF-b1), and hepatocyte growth factor (HGF) are also responsible for the observed inhibitory properties of BMSCs [15–17]. More importantly, indoleamine 2,3-dioxygenase (IDO), and inducible NO synthase (iNOS), have been shown to be produced by BMSCs and function as critical local immune suppressive factors in the prevention of immunological rejection[18,19].In addition, phenotypic analysis revealed that BMSCs were positive forCD73, CD90, CD105, and MHC I. Meanwhile, they were negative for hematopoietic markers (CD34) and surface proteins involved in immunological signal transduction, such as MHC II and co-stimulatory molecules (CD40, CD80 andCD86). This surface antigen pattern may be in part responsible for the immuno-privileged status of BMSCs [20].

Accordingly, the objectives of this study were to examine the immunogenicity and immunomodulatory capacities of dermal BMPR-IB^+^ cells before and after osteogenic differentiation compared with bone marrow mesenchymal stromal cells in a mouse model and explore the underlying mechanisms using allogeneic mixed lymphocyte reaction assay (MLR) *in vitro*.

## Materials and Methods

### Cells isolation and culture

4–6-week-old male C57BL/6 and Balb/c mouse were obtained from Shanghai Slac Laboratory Animal Center. Shanghai Ninth people’s Hospital Ethics committee approved the research and all animal procedures were performed to minimize any pain for animals.

#### Dermal BMPRIB^+^ fibroblasts

Dermal BMPRIB^+^ fibroblasts were isolated using the method as described previously [3]. Briefly, The abdominal skin specimens were digested with 8 U/ml dispase I(Worthington Biochemical Corporation, Lakewood, NJ, USA) at 4°C overnight. The separated dermis was further enzymatically digested at 37°C for 3 h with 375 U/ml type I collagenase (Sigma-Aldrich, St.Louis, MO, USA) in Dulbecco’s modified Eagle’s medium–low glucose (DMEM-Ig) (Gibco, Grand Island, NY,USA). The cell suspensions were washed and cultured in DMEM-Ig containing 10% fetal bovine serum (FBS) (Invitrogen Corporation, Carlsbad, CA), 100 U/ml penicillin and100 μg/ml streptomycin (all from Sigma, St Louis, MO)at 37°C in a humidified atmosphere containing 95% air and 5% CO_2_. The resulting fibroblastoid adherent cells were termed as dermal fibroblasts (FBs). When adherent cells were grown to 90% confluence, they were harvested at subconfluence using 0.25% trypsin/EDTA (Invitrogen).Cells of passage three were used for cell sorting. Appropriate 1X 10^7^/ml FBs were incubated with saturating concentrations (1:100dilution) of primary rabbit anti-mouse IgG monoclonal antibody BMPRIB (Santa cruz) for 20 minutes on ice, and then the cells were magnetically labeled with Anti-rabbit IgG microbeads. The BMPRIB-positive cells were sorted according to the manufacturer’s instructions (Mitenyi Biotec Inc, USA). The magnetic-activated cell sorting (MACS) procedure was generally repeated three times by using a new column to increase the purity of magnetically labeled cells.

The sorted populations were then analyzed using flow cytometry to determine the sorting efficiency. Briefly, cells were initially incubated with saturated (1:100) non-labeled anti-BMPRIB rabbit monoclonal antibody (Santa cruz) on ice for 20 min. After three washes, FITC-conjugated goat anti-rabbit IgG (1:100; abcam,Cambridge, UK)were used to amplify the signals. Cell suspensions were washed twice with PBS and resuspended in PBS containing2% FBS for analysis on a flow cytometer (FACS Calibur;BD Biosciences) using the CellQuest software. Only subpopulation with over 95% enrichment were cultured and used for subsequent studies.

#### Bone marrow mesenchymal stromal cells

Bone marrow mesenchymal stromal cells (BMSCs) were isolated from femurs and tibias of C57BL/6 and Balb/c mice using the plastic adherence method as described previously[6].Briefly, following femoral and tibial aspiration, the resulting cell suspensions were plated in DMEM-Ig containing 10% FBS, 100 U/ml penicillin and100 μg/ml streptomycin at 37°C in a humidified atmosphere containing 95% air and 5% CO_2_. Culture medium was changed every 3–4 days and cells were passaged using 0.25% trypsin/EDTA and replated at a low density of 1 × 10^3^ cells/cm^2^ into plates. The resulted fibroblast-like, adherent cells were called BMSCs. Cells of passage three were used for subsequent studies.

#### Splenocytes

Splenocytes were isolated from mouse spleens as described previously with minor variation [9].Briefly, spleens were carefully teased and passed through a cell sieve using Roswell Park Memorial Institute (RPMI)-1640 medium. Followed by red blood cell lysis with NH_4_Cl buffer solution(Sigma-Aldrich, St-Louis, MO), the resultant cell suspension was washed, counted using the trypan blue dye-exclusion method and resuspended inRPMI-1640 containing 10% FBS, 100 U/ml penicillin and100 μg/ml streptomycin for subsequent experiments.

### Osteogenic differentiation

At passage three, dermal BMPRIB^+^ cells and BMSCs were trypsinized and subcultured in 6-welltissue culture plates at a cell density of 1.0 ×10^3^/cm^2^. When cells grew to 90% confluence, the cells were cultured in osteogenic differentiation medium(DMEM-high glucose supplemented with 10%FBS, 1% antibiotics,0.01 μM 1,25-dihydroxyvitamin D3, 50 μM ascorbate-2-phosphate and 10 mM β-glycerophosphate) (all from Sigma) and renewed every 3 days. Cells grown in the original culture medium (DMEM-high glucose supplemented with 10%FBS and 1% antibiotics) were set as negative controls.

At 1-week, cells were stained for alkaline phosphatase (AP) activity according to manufactures’ protocols (Renbao, China).Quantification of AP activity was measured according to the protocol described previously [3]. After 2 weeks’ osteogenic induction, calcium deposition was analysed using alizarin red (AR) staining. Cells were fixed with 4% cold paraformaldehyde for 10 minutes, washed with distilled water and then stained with Alizarin Red solution (40 mM Alizarin Red-Tris-HCL, pH 4.1; Sigma) at room temperature for10 minutes. After three washes with distilled water, bone nodules staining positive for AR were counted under a microscope as described previously [3].

Meanwhile, osteogenic gene expression (Runx2, osteopontin, and osteocalcin) were also examined by quantitative real-time polymerasechain reaction (qRT-PCR) using an Applied Biosystems 7300 real-time PCRsystem and SYBR Green PCR Master Mix (Applied Biosystems). All values were normalized to ß-actin expression in the corresponding samples. The specific gene primers for mouse Runx2 (Mm00501580_m1), osteopontin (Mm00436767_m1), osteocalcin (Mm03413826_mH) and ß-actin (Mm00607939_s1) were purchased from Applied Biosystems.

Both undifferentiated and osteogenic differentiated cells were cultured in relevant medium for 14 days for subsequent studies [12].

### Phenotypic analysis

The expression of mesenchymal stem cell surface antigens (CD105, CD73, CD90 and CD34)(antibodies all from abcam, Cambridge, UK) and co-stimulatory molecules (MHC-I, MHC-II, CD80, CD86,andCD40) (antibodies all from eBioscience, UK) were analyzed by flow cytometry. Briefly, appropriate 0.5×10^6^ cells were washed and resuspended in 100μl of the buffer(PBS containing 2% FBS). Saturating concentrations (1:50) of fluorescein isothiocyanate (FITC)-conjugatedanti-CD105, anti-CD73,anti-CD90,anti-CD34, anti-CD80, anti-MHC-I, anti-MHC-II, or phycoerythrin (PE)-conjugated anti-CD40, anti-CD86were added into each of tubes and incubated on ice for 30 minutes. After two washes with PBS, all cell suspensions were resuspended in PBS containing 2% FBS for analysis on a flow cytometer (FACSCalibur; BD Biosciences) using the CellQuest software.

### Immunological assays

#### One-Way Mixed Lymphocyte Reaction (MLR, Immunogenicity Assay)

C57BL/6BMPRIB^+^fibroblasts and BMSCs were initially inactivated by treatment with 25ug/ml mitomycin C (sigma) at 37°C for 1 hour before and after the osteogenic differentiation. Then they were co-cultured with responder Balb/c splenocytes(1×10^5^ cells) at a 1:1 cell ratio in 200μl RPMI-1640 culture medium in 96-well plates. Co-culturing with stimulator C57BL/6splenocytesat a 1:1 cell ratio was used as positive control. Negative control was composed of Balb/c splenocytes only. The cultures were incubated for 5 days before measured by ^3^H-thymidine uptake. 5 uCi/ml^3^H-thymidine was added to each well 24 hours before harvest and the radioactivity was measured by a scintillation counter as counts per minute (CPM).

#### Two-Way Mixed Lymphocyte Reaction (Immunosuppression Assay)

Responder Balb/c splenocytes (1×10^5^ cells) were co-cultured with stimulator C57BL/6 splenocytes (1×10^5^ cells) in the presence of mitotically inactivated C57BL/6B MPRIB^+^ fibroblasts or BMSCs (undifferentiated or osteogenic differentiated). The added cell number ranged from1×10^4^, 5×10^4^ and 1×10^5^. Positive control comprised Balb/c splenocytes and C57BL/6 splenocytes only. Negative control comprised Balb/c splenocytes only. The cultures were incubated for 5 days before measured by ^3^H-thymidine uptake.

For iNOS or IDO inhibition assay, mitotically inactivated undifferentiated or osteogenic differentiated C57BL/6 BMPRIB^+^ fibroblasts(1×10^5^ cells) were co-cultured with Responder Balb/c splenocytes and stimulator C57BL/6 splenocytes at a 1:1:1 ratio. Meanwhile, the iNOS inhibitor N-[[3-(aminomethyl)phenyl]methyl]-ethanimidamide(1400 W, Cayman Chemical, Ann Arbor, MI, USA) or IDO inhibitor 1-methyl tryptophan (1-MT,Sigma, USA) was added to the culture medium at a gradient concentration (0.1mM, 1mM). The cells were cultured for 5 days and then proliferation assays were performed.

### Western blot analysis

Undifferentiated or osteogenic differentiated C57BL/6 BMPRIB^+^ fibroblasts were added to Responder Balb/c splenocytes (1×10^5^ cells) co-cultured with stimulator C57BL/6splenocytes at a 1:1:1 cell ratio for 5 days. C57BL/6 BMPRIB^+^ fibroblasts were then harvested to detect the expression of iNOS and IDO. Briefly, proteins were extracted with lysis buffer PMSF and then size-fractionated by sodium dodecyl sulfate–polyacrylamide gel electrophoresis (SDS-PAGE). After that, they were transferred to a nitrocellulose membrane and incubated with saturated concentrations of antibodies including anti-iNOS antibody (1:500) or anti-IDO antibody (1:50) (both from Abcam, Cambridge, UK).

### Statistical analysis

Data were expressed as mean± standard deviation from 3 independent experiments. ANOVA or paired t-test was applied to test statistically significant differences between observations. A value of P<0.05 was considered statistically significant. Calculations were performed using the SPSS 13.0 (SPSS, Chicago, IL, USA).

## Results

### Dermal BMPRIB^+^ cells possess a similar osteogenic differentiation capacity with BMSCs *in vitro*

**Fig 1:**
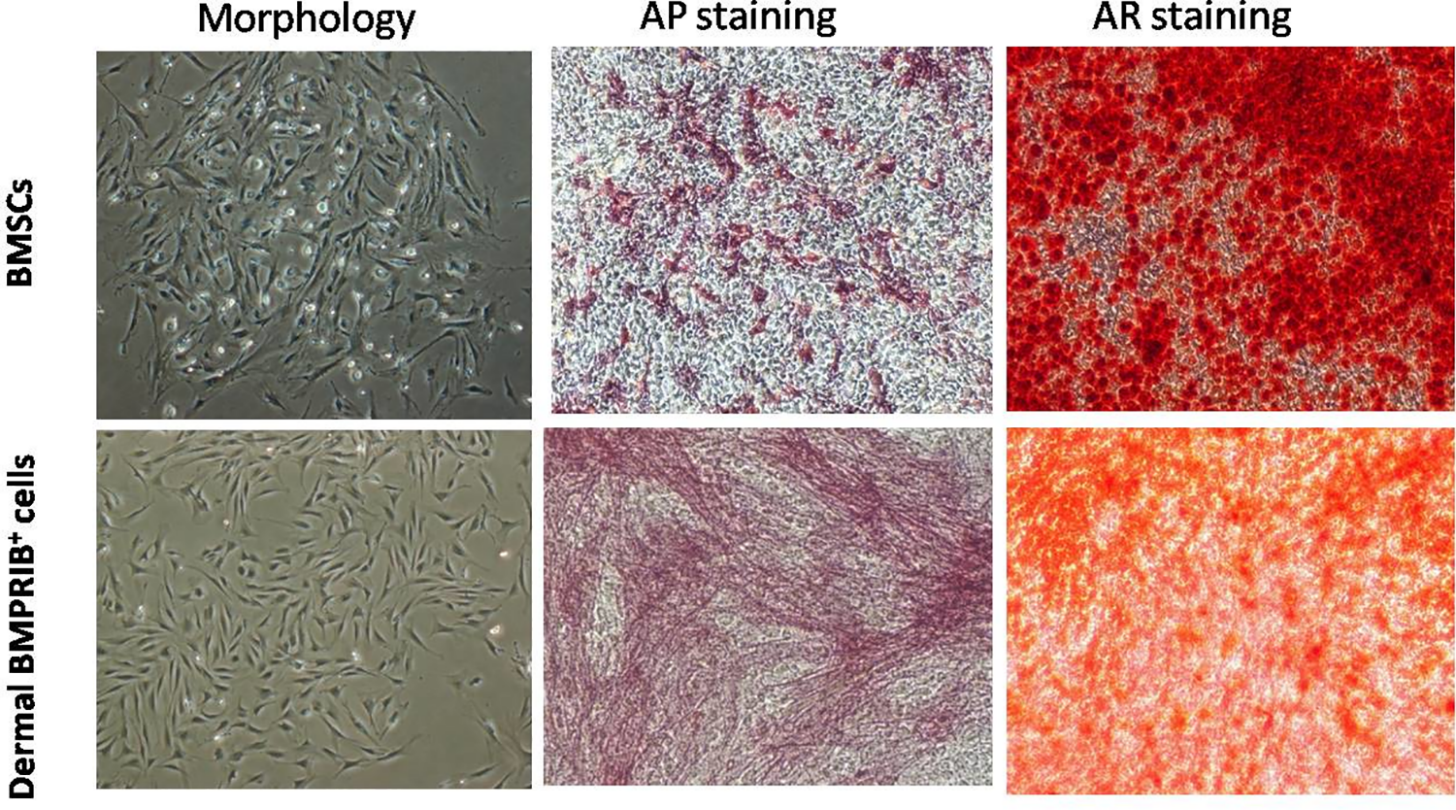
Characterisation and osteogenic differentiation of mouse dermal BMPRIB^+^ cells and BMSCs. Both the two cell types showed typical spindle-shaped morphology (×40) in regular cultures (*left*). After one week of osteogenic differentiation, a positive AP staining was detected in either dermal BMPRIB^+^ cells or BMSCs (*middle*). Moreover, they were stained positive for AR after two weeks’ induction (*Right*). In contrast, only very week AP staining or AR staining was observed when they were cultured in non-osteogenic conditions (images not shown).***BMPRIB***^***+***^ ***cells***: morphogenetic protein receptor IB positive cells, ***BMSCs*:** bone marrow mesenchymal stromal cells, ***AP*:** alkaline phosphatase, ***AR*:** Alizarin Red.

**Fig 2:**
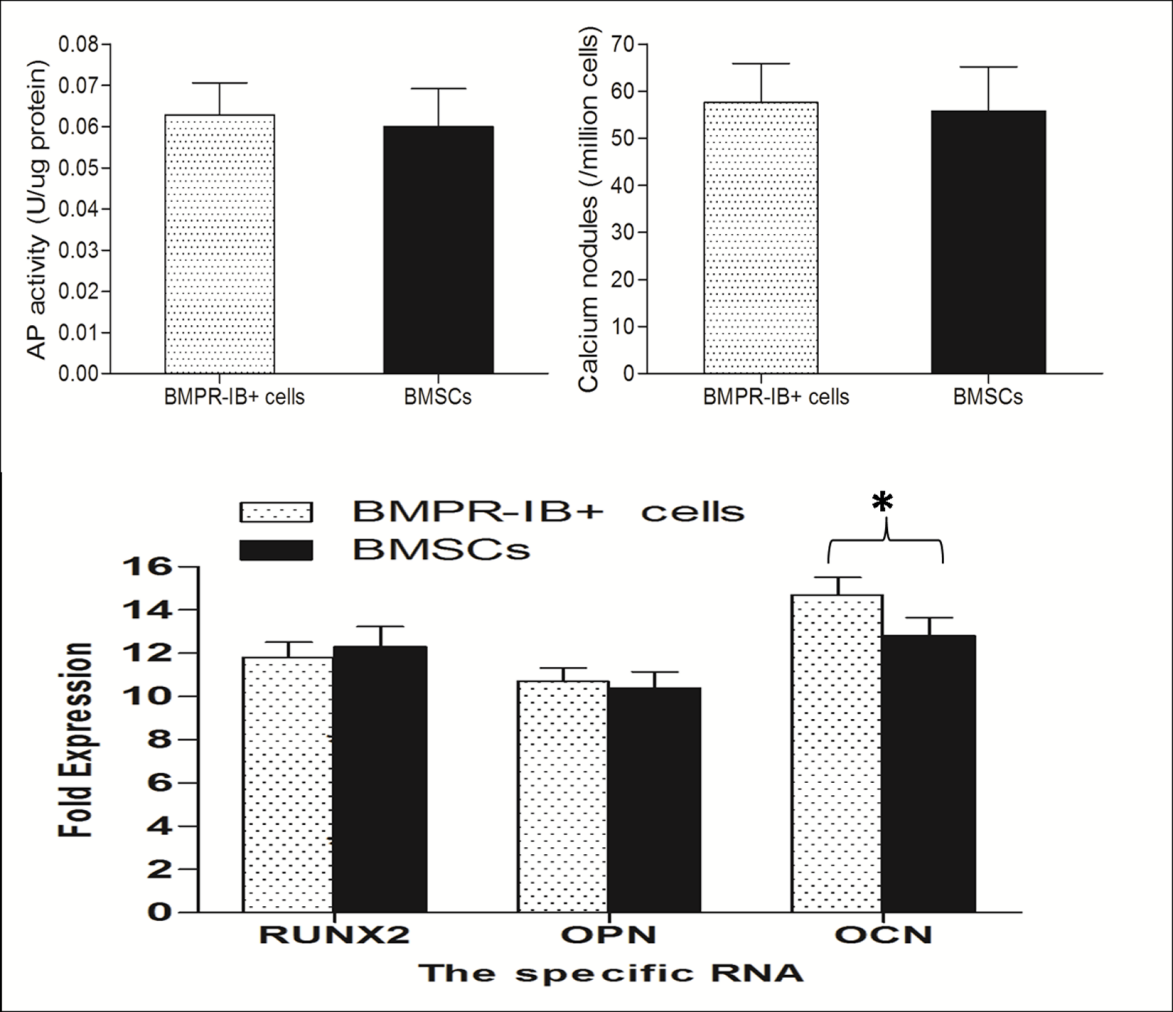
Comparision of osteogenic differentiation potential in mouse dermal BMPRIB^+^ cells and BMSCs. Under osteogenic conditions, dermal BMPRIB^+^ cells and BMSCs showed a similar AP production at one week *(above*, *left)* and calcium nodules formations at two week *(above*, *right)*. For osteogenic gene expression on mRNA level at day 14, dermal BMPRIB^+^ cells produced a significantly higher OCN (a late osteogenic marker) expression than BMSCs, while transcript levels for Runx2 (an early osteogenic marker)and OPN (an intermediate osteogenic marker)were not significantly different between two groups *(below)*.Bars represent mean ± SD of n = 3 donor samples.^**＊**^p<0.05. ***BMPRIB***^***+***^ ***cells***: morphogenetic protein receptor IB positive cells; ***BMSCs*:** bone marrow mesenchymal stromal cells; ***OPN*:** osteopontin; ***OCN*:** osteocalcin.

Given the previous work by our laboratory, we employed magnetic cell sorting to successfully isolate BMPRIB^+^ cells in mouse dermis. Through flow cytometry analysis, we demonstrated that MACS sorting resulted in BMPRIB^+^ subpopulations with over 95% enrichment. Under regular cultures, dermal BMPRIB^+^ cells and BMSCs exhibited a similar elongated and spindled-shaped morphology during the assay period. After one week of osteogenic induction, an osteoblast-like appearance was detected and stained positive for AP for both cell types. Moreover, quantification of AP activity showed a similar AP production between dermal BMPRIB^+^ cells group and BMSCs group. After 14 days in osteogenic differentiation medium, Alizarin red staining was further performed to detect extracellular matrix mineralization. Similar to that observed with AP staining, a considerable amount of bone nodules were detected for both groups (Fig 1). In addition, we also examined osteogenic gene expression on mRNA level at day 14 and found that dermal BMPRIB^+^ cells produced a significantly higher osteocalcin(OCN, a late osteogenic marker) expression than BMSCs, while transcript levels for Runx2(an early osteogenic marker)and osteopontin (OPN, an intermediate osteogenic marker)were not significantly different between two groups (Fig 2). Collectively, the osteogenic differentiation capacity was successfully achieved in cells from both sources.

### Characteristics of immunologically relevant surface antigens in BMPRIB^+^ cells

Phenotypic analysis of BMPRIB^+^ cells and BMSCs at passage three was performed by flow cytometry. For mesenchymal stem cell related surface markers, BMPRIB^+^ cells and BMSCs were strongly positive for CD73 (89±2.2% *VS*.90±1.3%) and CD90 (91±1.4% *VS*. 85±1.9%), but negative for CD34 (1.8±0.2% *VS*.3.1±1.1%). BMPRIB^+^ cells were moderately positive for CD105 (52±1.9%) compared with BMSCs (87±2.7%). For immunologically relevant surface antigens, BMPRIB^+^ cells and BMSCs showed positive reactivity for MHC class I molecules, but lacked expression of MHC class II molecules and co-stimulatory molecules (CD80, CD86, and CD40).After osteogenic differentiation *in vitro* for two weeks, there was no significant variation on the expression of these immunologically relevant surface antigens for cells from the two sources (Table 1).

**Table 1:** Phenotypic analysis of C57BL/6 BMPRIB^+^ cells and BMSCs before and after osteogenic differentiation by flow cytometry.

Marker	BMPRIB^+^ cells		BMSCs	
	undifferentiated	differentiated	undifferentiated	differentiated
**MHC I**	87±1.2%	66±2.3%	90±2.1%	70±2.7%
**MHC II**	3.4±0.9%	1.1±1.3%	2.8±0.6%	1.4±0.5%
**CD80**	2.5±1.9%	1.7±0.5%	3.6±0.3%	1.5±0.7%
**CD86**	4.1±0.8%	1.4±1.0%	4.4±0.2%	1.8±0.7%
**CD40**	2.4±0.9%	3.5±1.1%	2.9±0.6%	3.4±0.9%

BMPRIB^+^ cells: morphogenetic protein receptor IB positive cells; BMSCs: bone marrow mesenchymal stromal cells; MHC I: major histocompatibility class I; MHC II: major histocompatibility class II. The percent of positively stained cells relative to isotype controls was expressed in the results.

### Differentiated and undifferentiated BMPRIB^+^ cells do not elicit allogeneic splenocyte proliferation

**Fig 3:**
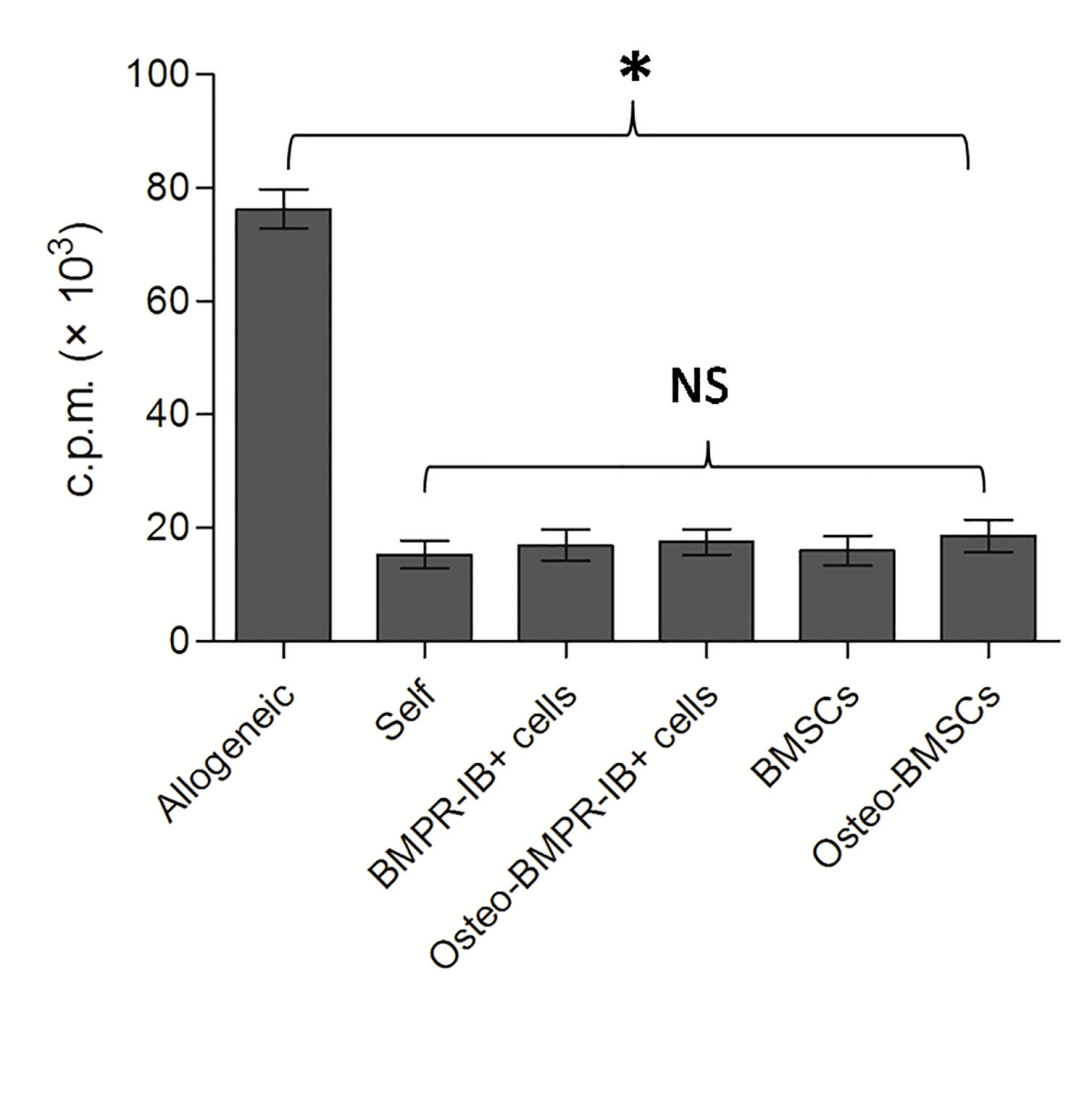
Immunogenicity of C57BL/6 BMPRIB^+^ cells and BMSCs by one-way mixed lymphocyte reaction assay. Balb/c splenocytes were co-cultured with four mitotically inactivated different cell isolates at passage three at a cell ratio of 1:1for 5 days. Autologous mitotically inactivated splenocytes were set as negative control, while C57BL/6 mitotically inactivated splenocytes were set as positive control. Bars represent mean ± SD of n = 3donor samples.^**＊**^p<0.05. ***BMPRIB***^***+***^ ***cells***: morphogenetic protein receptor IB positive cells; ***BMSCs*:** bone marrow mesenchymal stromal cells; ***NS*:** no significant.

To determine the immunogenicity of differentiated and undifferentiated BMPRIB^+^ cells *in vitro*, the mitotically inactivated cells were co-cultured with allogeneic splenocytes at a ratio of 1:1 for 5 days. As indicated in Fig 3, allogeneic BMPRIB^+^ cells and BMSCs did not produce significantly higher counts than syngeneic splenocyte group, while a significant higher proliferation was detected in allogeneic splenocyte group. The results demonstrated that BMPRIB^+^ cells and BMSCs were immunoprivileged as they did not elicit allogeneic splenocyte proliferation *in vitro*. Furthermore, after two weeks culture under osteogenic inducing conditions, the differentiated BMPRIB^+^ cells and BMSCs also produced comparable counts to syngeneic splenocyte group, indicating that osteogenic differentiated BMPRIB^+^ cells and BMSCs failed to stimulate proliferation response *in vitro*. Osteogenic differentiation did not have an adverse effect on the allogeneic splenocyte proliferation.

### Differentiated and undifferentiated BMPRIB^+^ cells possessed immunosuppressive effect on the allogeneic MLR

**Fig 4:**
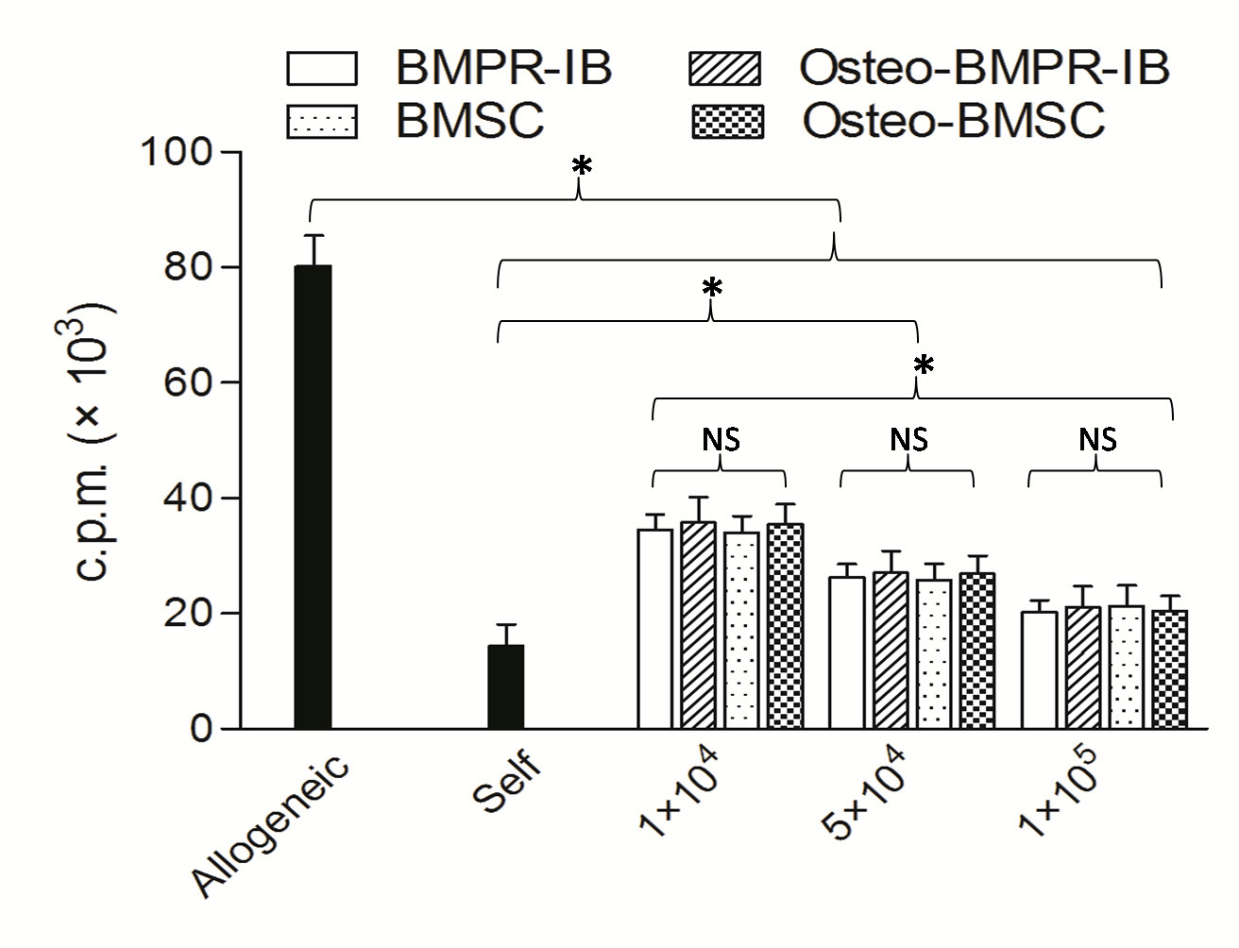
Immunosuppressive capacity of C57BL/6 BMPRIB^+^ cells and BMSCs by two-way mixed lymphocyte reaction (MLR) assay. Four mitotically inactivated different cell isolates were added to allogeneic MLR at the numbers ranging from 10000, 50000, and 100000 cells per well. Autologous inactivated splenocytes were set as negative control. Bars represent mean ± SD of n = 3donor samples.^**＊**^p<0.05. ***BMPRIB***^***+***^ ***cells***: morphogenetic protein receptor IB positive cells; ***BMSCs*:** bone marrow mesenchymal stromal cells. ***NS*:** no significant.

To investigate possible immunosuppressive properties of differentiated and undifferentiated BMPRIB^+^ fibroblasts, the mitotically inactivated cells were added as third parties with gradient concentration to responder cells (Balb/c splenocytes)co-incubated with inactivated stimulator cells (C57BL/6splenocytes). As showed in Fig 4, when increasing numbers of BMPRIB^+^ cells or BMSCs were added to the allogeneic MLR, a great suppression of MLR response was observed in a dose dependent manner. A ratio of 1 BMPRIB^+^ cells or BMSC to 1 PBMNC almost completely suppressed allogeneic MLR response for 5 days. More importantly, osteogenic differentiated BMPRIB^+^ cells or BMSCs also inhibited the allogeneic MLR response at similar levels to that observed for undifferentiated cells.

### Mechanisms mediating immunomodulatory effects of differentiated and undifferentiated BMPRIB^+^ cells on allogeneic splenocyte proliferation

**Fig 5:**
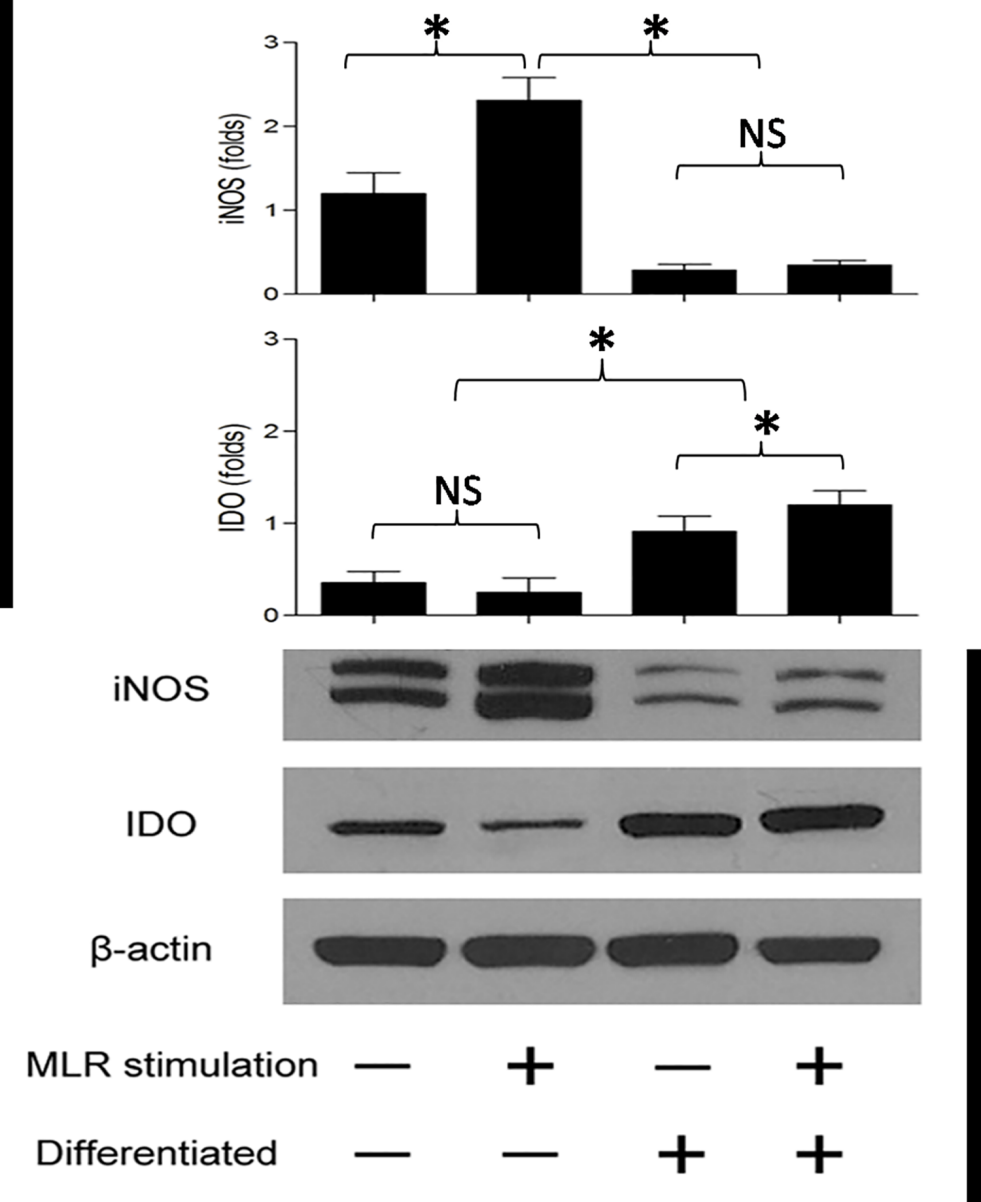
Mechanisms mediating immunosuppressive capacity of C57BL/6 BMPRIB^+^ cells by western blot analysis. Following co-culture with allogeneic MLR for 5 days, undifferentiated BMPRIB^+^ cells upregulated the iNOS expression drastically above the basal level, while IDO production was found to be upregulated greatly in osteogenic BMPRIB^+^ cells. Bars represent mean ± SD of n = 3donor samples.^**＊**^p<0.05. ***BMPRIB***^***+***^ ***cells*:** morphogenetic protein receptor IB positive cells; ***MLR*:** mixed lymphocyte reaction; ***iNOS*:** inducible NO synthase; ***IDO*:** indoleamine 2,3-dioxygenase; ***NS*:** no significant.

**Fig 6:**
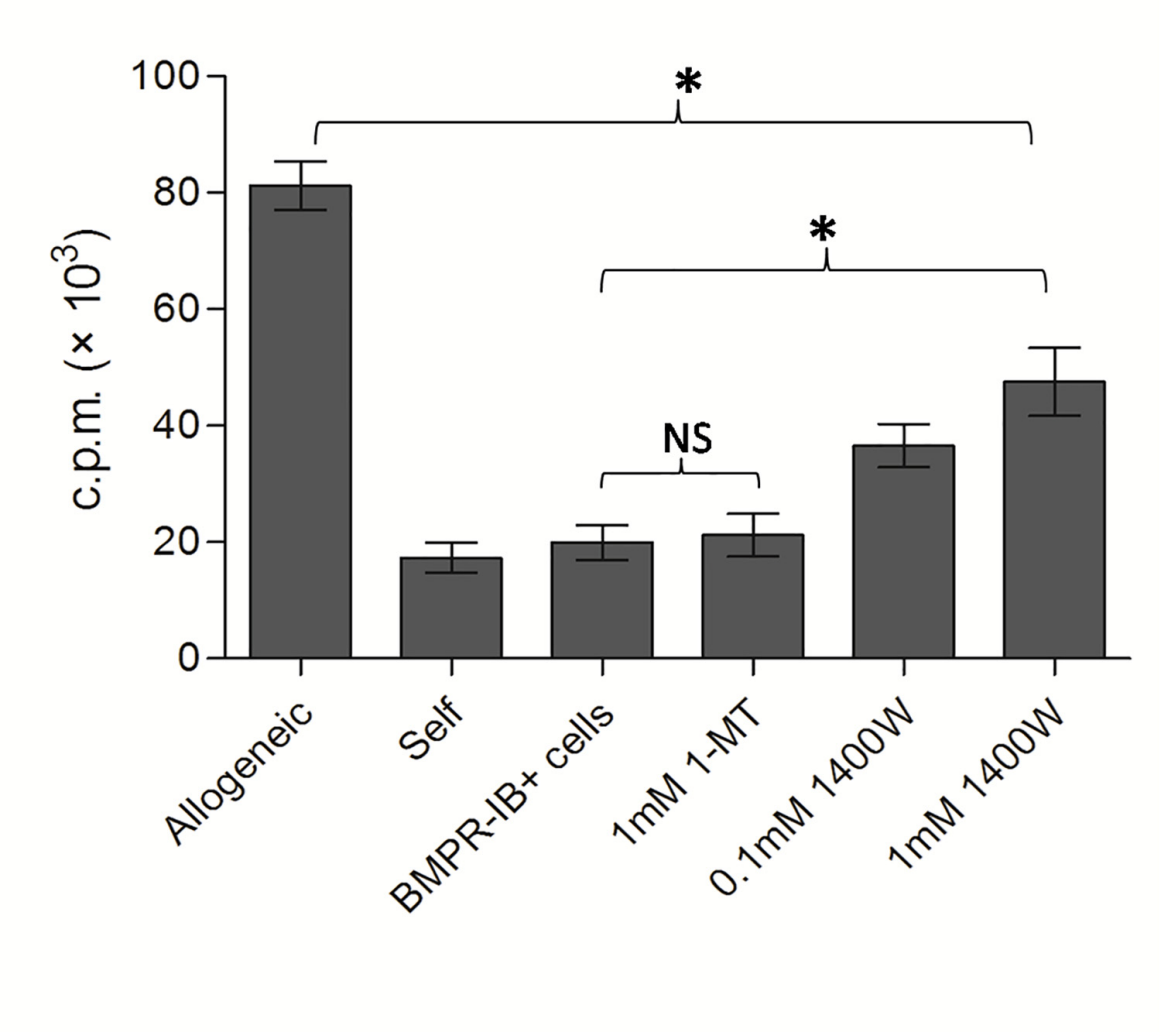
Selective iNOS blockade with 1400W partly reverses the effect of dermal BMPRIB^+^ cells on allogeneic splenocytes proliferation. Mitotically inactivated undifferentiated C57BL/6 BMPRIB^+^cells (1×10^5^ cells) were co-cultured with Responder Balb/c splenocytes and stimulator C57BL/6 splenocytes at a 1:1:1 ratio in 200μl culture medium. Cells were treated with 1400W (0.1mM, 1mM) or 1-MT (1mM) for 5 days and proliferation was performed respectively. Bars represent mean ± SD of n = 3donor samples.^＊^p<0.05. ***BMPRIB*^*+*^*cells***: morphogenetic protein receptor IB positive cells; ***1400W***: N-[[3-(aminomethyl)phenyl]methyl]-ethanimidamide; ***1-MT***:1-methyl tryptophan. ***NS***: no significant.

Several studies have demonstrated that soluble factors, such as iNOS and IDO were secreted by BMSCs and play critical roles in mediating their immunoregulatory effects (18, 19).To determine whether the immunosuppressive properties of BMPRIB^+^ cells were mediated via the secretion of inhibitory factors, we further measured the expression of iNOS and IDO in BMPRIB^+^ cells before and after the osteogenic differentiation. The results showed that iNOS expression in undifferentiated BMPRIB^+^ cells was significantly higher than that in osteogenic differentiated BMPRIB^+^ cells. Following co-culture with allogeneic splenocytes for 5 days, the iNOS expression was upregulated drastically above the basal level in undifferentiated BMPRIB^+^ cells (Fig 5).More importantly, selective iNOS blockade with 1400W in undifferentiated BMPRIB^+^ cells caused an obvious dose-dependent restoration in allogeneic splenocyte proliferation, while an addition of INO inhibitor,1-methyl tryptophan, failed to restore the allogeneic proliferation (Fig 6). Consequently, iNOS secreted by BMPRIB^+^ cells play a critical role in mediating immunosuppressive effects. By contrast, no obvious variation of iNOS expression was detected for osteogenic differentiated BMPRIB^+^ cells when co-cultured with allogeneic splenocytes.

**Fig 7:**
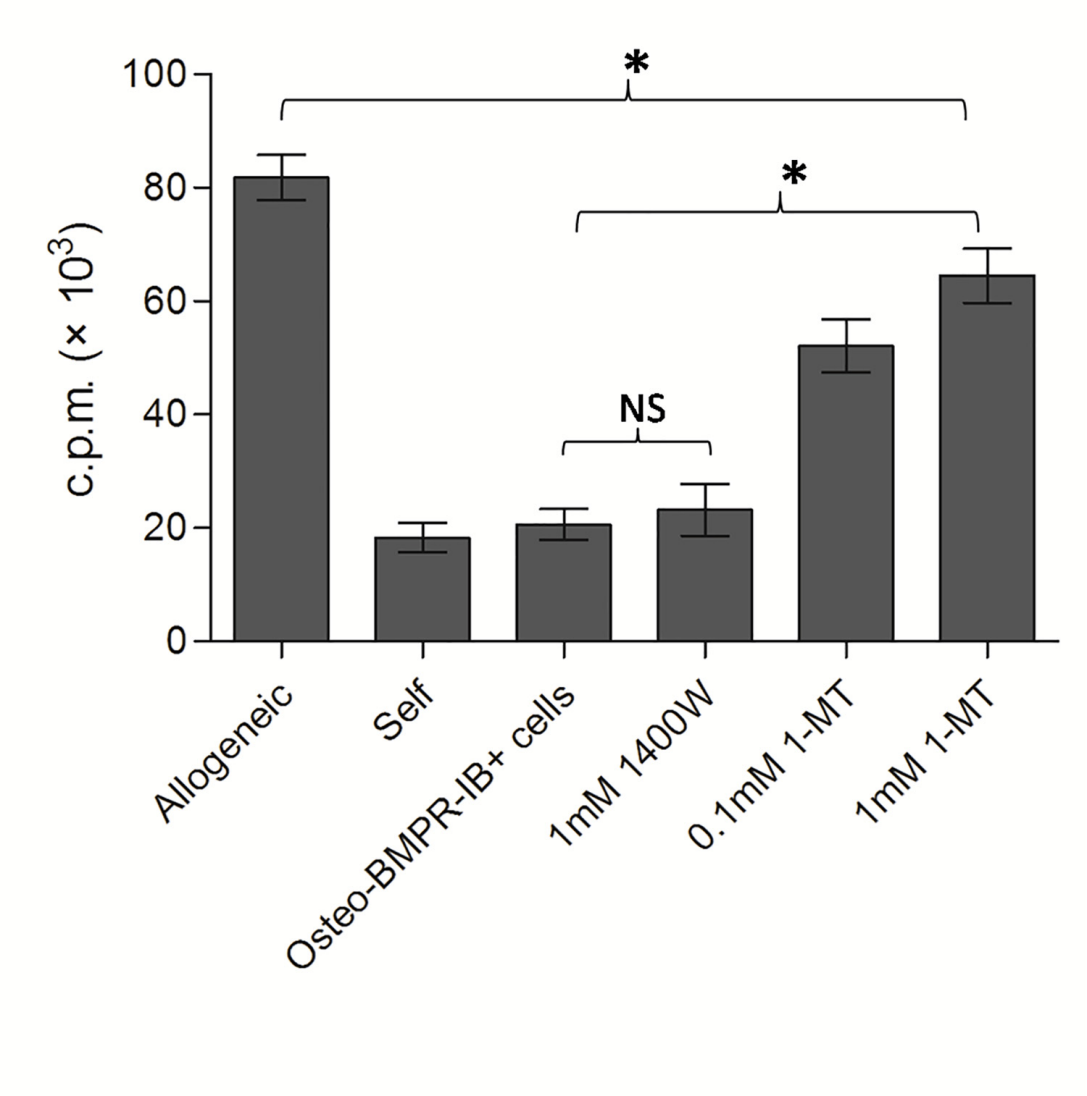
IDO inhibitor 1-MT effectively restores the allogeneic splenocytes suppression induced by osteogenic differentiated BMPRIB^+^ cells. Mitotically inactivated osteogenic differentiated C57BL/6 BMPRIB^+^ cells (1×10^5^ cells) were co-cultured with Responder Balb/c splenocytes and stimulator C57BL/6 splenocytes at a 1:1:1 ratio in 200μl culture medium. Cells were treated with 1-MT (0.1mM, 1mM) or 1400W (1mM) for 5 days and proliferation was performed respectively. Bars represent mean ± SD of n = 3donor samples.^**＊**^p<0.05. ***BMPRIB***^***+***^ ***cells*:** morphogenetic protein receptor IB positive cells; ***1400W*:** N-[[3-(aminomethyl)phenyl]methyl]-ethanimidamide; ***1-MT*:**1-methyl tryptophan. ***NS*:** no significant.

The IDO expression was also detected by western blot to explore the mechanism of suppressive effect caused by osteogenic differentiated BMPRIB^+^ cells. As can be clearly seen in Fig 5, osteogenic differentiated BMPRIB^+^ cells constitutively express IDO in a very low level, but a substantial amount of IDO protein is induced by co-culturing with allogeneic splenocytes. To confirm whether IDO expression directly mediates the suppression of allogeneic splenocyte proliferation, 1-methyl tryptophan, a specific inhibitor of IDO, was added to the culture medium at a gradient concentration. As expected, allogeneic splenocyte proliferation was greatly recovered in the treated medium (Fig 7). The results suggested that IDO played an important role in mediating immunosuppressive property of osteogenic differentiated BMPRIB^+^ cells.

## Discussion

In this study, we confirmed that dermal BMPRIB^+^ subpopulations could be isolated in large quantities and possessed a similar osteogenic differentiation potential with BMSCs in a mouse model. Furthermore, the immune properties of dermal BMPRIB^+^ subpopulations were described for the first time. Flow cytometry revealed that the expression of immune rejection-related surface antigens MHC II and co-stimulatory proteins (CD40, CD80, and CD86) were absent on dermal BMPRIB^+^ cells. Dermal BMPRIB^+^ cells elicited no proliferation of allogeneic splenocytes and suppressed the proliferation of stimulated immune cells. Additionally, osteogenic differentiation *in vitro* had no adverse effect on the immunological features of these cells. Interestingly, iNOS was involved in immunoregulatory effects by undifferentiated BMPRIB^+^ fibroblasts, whereas IDO activity was related to mediating immunomodulatory function by osteogenic differentiated BMPRIB^+^ fibroblasts.

Generally, the immunomodulatory capacity is believed to be the primitive stem cell function and adult stromal cells such as dermal fibroblasts do not possess immunosuppressive properties [11]. However, Haniffa MA et al. reported that dermal fibroblasts shared the immunosuppressive effects of MSCs and could inhibit allogeneic T cells activation by autologously derived antigen-presenting cells. The IDO expression on dermal fibroblasts was partly responsible for the suppression of T cells proliferation [21]. Wada N et al. revealed that human foreskin fibroblasts expressed an MSC-like immunophenotype and suppressed human peripheral blood mononuclear cells proliferation stimulated with mitogen comparable to BMSCs [22].As the dermis comprises a variety of heterogeneous cell types, we further isolated BMPRIB^+^ subpopulations from dermis in our study. The results showed that dermal BMPRIB^+^ subpopulations were also able to suppress allogeneic lymphocytes *in vitro* similar to bone marrow mesenchymal stromal cells. Immunoregulation maybe an intrinsic property of adult stromal cells and dermal BMPRIB^+^ subpopulations are promising alternative source for allogeneic bone tissue engineering.

The potential clinical application would also take the form of osteogenic differentiation of allogeneic dermal BMPRIB^+^ cells. Thus, it is necessary to investigate the impact of osteogenic differentiation on their immunogenicity and immunosuppressive properties. Our flow cytometry analysis revealed that the differentiated dermal BMPRIB^+^ cells displayed no expression of MHC II on their cell membranes as well as co-stimulatory molecules, such as CD80, CD86 and CD40. Obviously, the immunologically surface protein profiles were in part responsible for the low immunogenicity of differentiated dermal BMPRIB^+^ cells. Moreover, differentiated dermal BMPRIB^+^ cells also suppressed the allogeneic splenocytes proliferation *in vitro*, as demonstrated in our results.

Previous studies by various groups have showed that immunosuppressive function of MSCs is not innate, but is induced by several cytokines. Upon stimulation, MSCs could upregulate the iNOS and IDO expressions dramatically. These cytokines were functionally strong local immunosuppressive factors to prevent allogeneic immune cell rejection [11, 23]. To explore insights into the mechanisms of immunomodulatory capacity of dermal BMPRIB^+^ subpopulations before and after osteogenic differentiation, we further evaluated iNOS and IDO expressions in dermal BMPRIB^+^ cells under the stimulation with allogeneic MLR. Our western blot analysis found that a dramatic increase of iNOS above the basal level was detected in undifferentiated dermal BMPRIB^+^ cells following co-culture with allogeneic MLR. Selective iNOS blockade with 1400W led to a marked dose-dependent restoration in allogeneic splenocyte proliferation. The results showed that iNOS played a critical role in mediating the immunosuppression in undifferentiated dermal BMPRIB^+^ cells.

However, after osteogenic differentiation, we further demonstrated that a substantial amount of IDO protein is induced in dermal BMPRIB^+^ cells by co-culturing with allogeneic splenocytes. The addition of IDO inhibitor, 1-MT, could effectively recover the allogeneic splenocytes proliferation, indicating that IDO played a role in mediating immunosuppression in differentiated dermal BMPRIB^+^ cells.

Collectively, these data preliminary showed that although both undifferentiated and osteogenic differentiated dermal BMPRIB^+^ cells possessed immunosuppressive properties *in vitro*, the mechanisms of immunosuppression in two cell types were mediated through the production of different cytokines after stimulation with allogeneic MLR. Further studies should lead to a thorough understanding of this phenomenon.

Although osteogenic differentiated dermal BMPRIB^+^ cells had immunosuppressive properties *in vitro*, we should also be concerned about the potential allogeneic immune rejection *in vivo* when applying them in cell-based tissue replacement therapies. Currently, several studies have proved that MSCs derived from bone marrow or adipose tissue maintained their immunologic profiles before and after differentiation *in vitro*[6, 12]. However, contradictory reparative outcomes in animal models in relation to the immunogenicity of osteogenic differentiated MSCs have also been reported [13]. Additionally, it is not clear that the osteogenic differentiation for 2 weeks *in vitro* could lead to fully differentiation of dermal BMPRIB^+^ cells despite the matrix synthesis in terms of mineralization was demonstrated using Alizarin Red staining. Quite a few allogeneic dermal BMPRIB^+^ cells may be still in an undifferentiated state and suppress the allogeneic splenocytes proliferation. As dermal BMPRIB^+^ cells are easily accessible, it is significant to further explore the in vivo efficacy of dermal BMPRIB^+^ cells-mediated immunosuppression.

In conclusion, dermal BMPRIB^+^ cells possessed a similar osteogenic differentiation potential with BMSCs in a mouse model. Dermal BMPRIB^+^ subpopulations possessed low immunogenicity and immunosuppressive properties before and after osteogenic differentiation. Interestingly, iNOS was involved in immunoregulatory effects by undifferentiated BMPRIB^+^ cells, whereas IDO activity was related to mediating immunomodulatory function by osteogenic differentiated BMPRIB^+^ cells.
